# Cloning and Recombinant Expression of the Caspase-Activated DNase Orthologous Gene of *Giardia lamblia*

**DOI:** 10.1155/bmri/3420875

**Published:** 2025-03-06

**Authors:** María Cristina Villa-Medina, Cecilia Díaz-Gaxiola, Roberto Rosales-Reyes, Sergio Alonso Durán-Pérez, Ulises Vega-Castillo, Jesús Alberto Rodríguez-Rochín, Claudia del Rosario León-Sicairos, Evangelina Beltrán-López, Héctor Samuel López-Moreno

**Affiliations:** ^1^Lab. Biomedicina Molecular, Posgrado en Ciencias Biomédicas, CAC-UAS-264, Facultad de Ciencias Químico Biológicas, Universidad Autónoma de Sinaloa, Culiacán, Sinaloa, Mexico; ^2^Unidad de Medicina Experimental, Hospital General de México, Universidad Nacional Autónoma de México, Mexico City, Mexico

**Keywords:** apoptosis, caspase-activated DNase, *Giardia lamblia*, human CAD, recombinant protein

## Abstract

In eukaryotic cells, mitochondria play a key role in apoptosis; however, ancestral eukaryotic cells such as *Giardia lamblia* only possess a mitochondrial remnant, the mitosome. Interestingly, this protozoan still undergoes an apoptosis-like process; therefore, we focused primarily on the search for the mitochondria-independent executor DNase. Here, we identified, cloned, expressed, and characterized the caspase-activated DNase (CAD) from *Giardia lamblia*. Using a commercial polyclonal antibody that recognizes mouse, rat, and human caspase-activated DNase (hCAD), we developed an immunoproteomic analysis using a crude extract of curcumin-treated *Giardia lamblia* trophozoites (CEGl) and detected a spot of 42 kDa and pI 9.4, similar to hCAD and sequenced by LC-MS. The proteomic profile matched a novel protein of 383 residues, with a predicted 42 kDa, pI 9.4, a CIDE-N domain, and putative H-K-H catalytic motif. Afterward, we cloned the full-length gene (GenBank: ON707040), expressed it, and purified it as a 6-His tag-recombinant protein in *Escherichia coli*, which was also recognized by commercial anti-CAD. In conclusion, genetic, proteomic, and structural analyses showed that the identified gCAD is an orthologous protein of hCAD, and its DNase role in the apoptosis-like signaling pathway of *Giardia lamblia* can be further analyzed.

## 1. Introduction

Apoptosis was described 50 years ago as a distinctive type of programmed cell death [1, 2], in which apoptotic cells suffer shrinkage and pyknosis [2] while maintaining membrane integrity. Notably, during the process, the cell flips inner phosphatidylserine (PS) to the outer leaflet of the membrane and degenerates into several smaller structures with tightly packed organelles called apoptotic bodies [1]. In mammals, macrophages remove these apoptotic bodies by PS-CD36 recognition [3]. The extracellular expression of PS is used to detect apoptosis in vitro through the interaction with annexin V conjugated to a fluorochrome or peroxidase [4]. In mammals, apoptosis can be activated by three pathways: (1) intrinsic pathway induced by cellular stress which involves mitochondrial activity and the apoptosome (a proteinic complex); (2) extrinsic pathway that involves tumoral necrosis factor receptor (TNFR) and the cysteine proteases family or caspases; and (3) perforin/granzyme pathway, induced by the immune response of effector CD8 T cells or natural killer (NK) lymphocytes [1]. These pathways converge in a downstream executory process that involves the liberation of caspase-activated DNase (CAD) from its inhibitor (ICAD), CAD is translocated to the nucleus, and DNA fragmentation is executed [1, 5–9].

The flagellated protozoan *Giardia lamblia* (synonyms: *Giardia intestinalis* or *Giardia duodenalis*) is a major cause of diarrheal diseases worldwide [10, 11]. As a eukaryotic cell, *Giardia lamblia* is considered an ancestral cell because it does not possess mitochondria besides a remnant body called mitosome [12, 13]. However, a report evaluating curcumin (derivate of *Curcuma longa*) as a pharmacological alternative against *Giardia lamblia* indirectly revealed that the protozoan employs apoptosis mechanisms, as suggested by results of annexin V staining results and DNA laddering [14]. In the mitochondria-independent apoptosis signaling pathway, CAD is involved in nuclear DNA fragmentation, after which its inhibitor (ICAD) is proteolytically removed by caspase-3 [5, 6, 9]. In eukaryotic cells, DNA fragmentation by CAD evokes an irreversible cellular change that ends in cell death by apoptosis [5, 7, 15, 16]. In this context, our objective was to identify, clone, and express CAD in *Giardia lamblia* (gCAD) to study and clarify the apoptosis-like signaling pathway of this protozoan. To achieve our goal, we performed an immunoproteomic assay to identify the amino acid sequence of the orthologous protein of gCAD curcumin-treated trophozoites. Subsequently, we cloned and expressed gCAD in a prokaryotic expression system that retained antigenicity. The gCAD structural and phylogenetic in silico analysis revealed conserved regions such as the cell death–inducing DFF45-like effector, N-terminal (CIDE-N) domain and H-K-H motif as possible catalytic domains, supporting the evidence that gCAD identified here is the first described protozoan CAD protein orthologous to human CAD.

## 2. Materials and Methods

### 2.1. Apoptosis-Like Induction in *Giardia lamblia*

Trophozoites of *Giardia lamblia* (ATCC 30957) were cultured on TYIS-33 media following the manufacturer's protocol. Since curcumin (*Curcuma longa*) (Sigma–Aldrich) has been described as a molecule that induces apoptosis-like death on *Giardia lamblia* trophozoites, we selected this agent as an apoptosis inductor on 3 × 10^5^ trophozoites (0, 0.3, 3.0, 30, and 100 *μ*M) for 24 h at 37°C, as previously described [14]. *Giardia lamblia* apoptosis-like death was evidenced by PS extracellular flip, which was detected with Alexa Fluor 488 annexin V/dead cell apoptosis kit, according to manufacturer instructions (ThermoFisher). The stained trophozoites were observed using a 100× objective in an immunofluorescence microscope (Nikon TE300); images were acquired with a digital camera (Hamamatsu) and analyzed using Metamorph software. Additionally, the treated trophozoites were observed by light inverted microscopy (Motic) using a 40× objective, and images were acquired with a digital camera 12 Mpx of an iPhone X (Apple).

### 2.2. Proteomic Analysis of *Giardia lamblia* Extracts

To identify gCAD, we used an immunoproteomic assay previously described by our group [17]. The cultures of 3 × 10^5^* Giardia lamblia* trophozoites were treated with curcumin 3 mM or untreated (as negative control) for 24 h at 37°C; the biomass was collected by centrifugation and was washed twice with PBS at pH 7.2. The biomass was resuspended in buffer containing 8 M urea, 2% CHAPS 2, 50 mM dithiothreitol (DTT), and a protease inhibitor cocktail (all from Sigma–Aldrich) and was lysed by three cycles of sonication for 30 s, with a separation of 30 s on ice. Lysates were centrifuged (10,000 × *g* for 15 min) at 4°C. The supernatants were collected and precipitated with eight volumes of cold acetone (JT Baker). Protein precipitates were washed three times with methanol (JT Baker). The protein concentration of the crude extract of curcumin-treated (or untreated) *Giardia lamblia* trophozoites (CEGl) was determined using the Bradford micromethod in ELISA plates (Bio-Rad). One hundred micrograms of CEGl was dissolved in 2D rehydration buffer containing 0.2% ampholytes and 0.002% bromophenol blue (Bio-Rad). CEGl was added to immobilized pH-gradient (IPG) 3–10 gel strips (Bio-Rad). The IPGs were incubated overnight at 20°C and resolved in a Protean IEF system (Bio-Rad) for 9 h. We used a program of 20 min at 250 V and 2 h at 4000 V, and the voltage was increased from 4000 to 10,000 V/h. Between the first and second dimensions, the IPGs were equilibrated in two steps: As the first step, each IPG was incubated for 15 min at room temperature (RT) in 375 mM Tris-HCl at pH 8.8, 6 M urea, 20% glycerol, 2% sodium dodecyl sulfate (SDS), and 2% DTT. As a second step, each IPG was incubated for an additional 15 min at RT in a second equilibrium buffer containing urea 333 mM, iodoacetamide 135 mM, SDS 2%, glycerol 20%, and Tris-HCl 375 mM pH 8.8. Individually, each IPG was 2D-resolved by duplicating on SDS-PAGE 12.5%, as previously described [17]; one was stained with Coomassie blue G-250 (Sigma–Aldrich) following standard protocols. The proteomic profile was digitalized on a photodocumentary system (Bio-Rad) and analyzed using Image-Lab software (Bio-Rad); the proteomic duplicated gel was used as an antigen for immunoproteomic analysis as described below [17].

### 2.3. Immunoproteomic Analysis of gCAD

With the duplicated 2D gel obtained, we performed Western blot analysis as described previously by our group with some modifications [17]. Briefly, the CEGl obtained from trophozoites treated or not treated with curcumin were electrotransferred on 0.2-*μ*m nitrocellulose membranes (Amersham) [17]; then, the membranes were blocked with skim milk 5% (Nestlé) in PBS supplemented with 0.1% Tween 20 (PBST) for 1 h at RT. The membranes were washed three times with PBST and incubated with a commercial polyclonal anti-CAD antibody (sc-8342, Santa Cruz Biotechnology) at 1:100 for 1 h at RT. [6] Membranes were washed three times using PBST and incubated with an anti-rabbit IgG horseradish peroxidase conjugate (BioLegend) at 1:3000 in PBST for 1 h, at RT. Afterward, the membranes were washed with PBST, the antigen–antibody reaction was confirmed using diaminobenzidine 0.05% (Research Organic) and H_2_O_2_ 0.05% (JT Baker) [17].

### 2.4. Identification of gCAD

The spot with the expected molecular weight (MW) and pI of gCAD was removed from the proteomic gel, transferred to a siliconized Eppendorf tube (Qiagen), and sent to the service unit of the Institute of Biotechnology (IBT), of the National Autonomous University of Mexico (UNAM), Cuernavaca, Mexico. They processed the samples as follows: DTT was used to reduce protein disulfide bonds, enabling more efficient digestion. Iodoacetamide was employed to modify cysteine residues, preventing their reoxidation and ensuring complete modification. Trypsin was used to fragment proteins into smaller peptides. The resulting peptides were separated using high-performance liquid chromatography (HPLC) on a capillary column packed with C18 material. An acetonitrile gradient allowed for the gradual elution of peptides. The eluted peptides were ionized by electrospray (ESI) and transferred to the mass spectrometer. The LTQ-Orbitrap Velos mass spectrometer acquired high-resolution mass spectra of the intact peptides. Precursor ions with 2^+^, 3^+^, and 4^+^ charges were selected for fragmentation using CID and HCD. The generated fragments provided information about the amino acid sequence of the peptides. The data obtained was compared to a *Giardia* protein database (UniProt) using the Proteome Discoverer 1.4 software. Proteins present in the sample were identified based on the correlation between experimental and theoretical spectra obtained from the database.

### 2.5. Recombinant Production of gCAD

With the predicted sequence of *gCAD* from GenBank sequence XM_001706846.1 and the software Oligo 7, Workbench, and Primer-BLAST, we designed specific primers to amplify the *gCAD* gene of *Giardia lamblia* GCAD-F 5⁣′-ATGCCAATCATCGTTAAGGGTG-3⁣′ and GCAD-R 5⁣′-TTACCTTGCCTCTTCGAGTGTC-3⁣′. Genomic DNA from *Giardia lamblia* was isolated using a DNeasy Blood & Tissue Kit (Qiagen). PCR was performed using PCR Master Mix (ThermoFisher) following the protocol: 95°C by 5 min, 30 cycles of 1 min at 94°C, 1 min at 60°C, and 1.5 min at 72°C, with a final extension step at 72°C, in a MiniAmp Thermal Cycler (ThermoFisher). The amplified *gCAD* gene was visualized in an agarose gel at 1% (*w*/*v*) and stained with SYBRGold (ThermoFisher). Afterward, the *gCAD* gene was purified using a NucleoSpin Gel and PCR Clean-up Kit (Macherey-Nagel). The *gCAD* gene was sequenced at the service unit of the Potosin Institute for Technological and Scientific Investigation (the IPICYT), San Luis Potosí, Mexico (deposited in GenBank: ON707040), and then cloned into the pET SUMO Champion prokaryote expression system (Invitrogen); the cloned sequence of *gCAD* was verified by sequencing at IPICYT service. Then, we expressed recombinant gCAD following the protocol reported previously by us [18]. Briefly, *Escherichia coli* BL21 carrying pgCAD was cultured in SOB medium supplemented with 100 mg/mL of kanamycin (Sigma), and when the optical density was 0.6 at 600 nm, gCAD expression was induced with Isopropyl *β*-D-1-thiogalactopyranoside (IPTG)1 mM for 7 h [18]. The biomass was collected by centrifugation at 5000 × *g* for 10 min at 4°C, and the pellet was resuspended in B-buffer (sodium phosphate 0.02 M, NaCl 1 M, pH 7.2) supplemented with protease inhibitor cocktail without EDTA (Sigma–Aldrich) to be lysed by sonication for 30 s; then, the sample containing the inclusion bodies was diluted 1:10 and solubilized with D-buffer (B-buffer plus hydrochloride guanidine 6 M, pH 7.2). The H_6_-tag-gCAD was purified by Ni-affinity chromatography on HiTrap chelating column (GE) as described previously [18]. The purified H_6_-tag-gCAD recombinant (rgCAD) was visualized using 12% SDS-PAGE and stained with Coomassie blue G-250 following standard protocols [18]. Finally, the immunorecognition of rgCAD by a commercial rabbit polyclonal anti-CAD was evaluated by Western blotting, as described in Section 2.3. All experiments were performed in triplicate.

### 2.6. Bioinformatics Analysis of gCAD

Until now, CAD detection in other protozoa has not been possible. To perform the phylogenetic analysis of gCAD, we compared different protein sequences and different species accessible in the National Center for Biotechnology Information (NCBI) database. A multiple sequence alignment (MAFFT v.7.487) [19] was performed between gCAD QAX90476.1 and organisms CAD: NP_001247612 (*Macaca mulatta*), XP_003891142.1 (*Papio anubis*), NP_004392 (*Homo sapiens*), NP_001020467 (*Mus musculus*), XP_417610 (*Gallus gallus*), XP_002939454 (*Xenopus tropicalis*), XP_003444949 (*Oreochromis niloticus*), and NP_001002631 (*Danio rerio*). Alignment was performed using the E-INS-I method [19]. A phylogenetic tree (neighbor-joining) was built with the latter complete aminoacidic sequences [17]. Data was analyzed using the Poisson replacement model, and the tree was visualized using PhyloXML [20] software and edited using FigTree. Bootstrap values were inferred from 1000 replicates and were used to represent the evolutionary history of the analyzed taxon. Finally, the prediction of the 3D structure of gCAD was carried out using AlphaFold [21] and edited with UCSF Chimera [22]. In addition, the 3D NMR structure of the CIDE-N domain of hCAD (Protein Data Bank (PDB) ID: 1IBX) and crystal structure of the CIDE-N domain of mCAD (PDB ID: ICF9) were used to compare with the CIDE-N domain of gCAD. Additionally, CLC Sequence Viewer 8.0 software was used to visualize the potential CIDE-N domain and catalytic motif of gCAD and to compare the results with those of hCAD and mCAD.

## 3. Results

### 3.1. Apoptosis of *Giardia*

**Figure 1:**
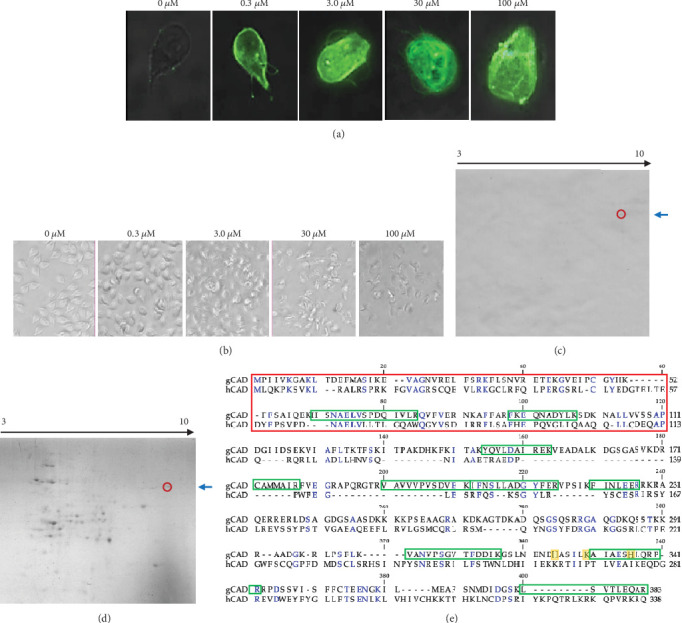
Identification of gCAD. (a) Trophozoites of *Giardia lamblia* were treated with different curcumin concentrations (0, 0.3, 3, 30, and 100 *μ*M) for 24 h and stained with annexin V-Alexa Fluor 488. Images were acquired using immunofluorescence microscopy (100×) coupled to a digital camera. (b) Trophozoites of *Giardia lamblia* were treated as in (a) and observed using light-inverted microscopy (40×). The images were acquired using an iPhone X camera. (c) Immunoproteomic detection of gCAD (pI 9.4 and 42 kDa) in 2D-SDS-PAGE followed by Western blotting; the gCAD localization is indicated with a blue arrow and red circle. (d) gCAD spot isolated the protein on a 2D SDS-PAGE Coomassie-stained gel of CEGl obtained as in (c) indicated with a blue arrow and red circle and thereafter sequenced by LC-MS. (e) The sequence of gCAD compared with hCAD. Identical amino acids are shown in blue, CIDE-N terminal domain is shown in red, and the putative catalytic motif (H-K-H) is highlighted in yellow. Proteomic peptide sequences (green rectangle) were obtained as in (d) and matched 100% with previously uncharacterized *Giardia lamblia* protein (GenBank XP_001706898.1). Here, we present the representative results of three independent experiments.

To detect the gCAD in apoptotic *Giardia lamblia*, we established the following apoptosis assay using trophozoites of *Giardia lamblia* incubated with curcumin for 24 h to expose the PS on its plasma membrane (Figure 1a), as demonstrated in eukaryotic cells that die by apoptosis [1, 2, 9, 23]. The following experiments were conducted using curcumin 3 *μ*M because higher concentrations induce dramatic changes in *Giardia*'s morphology (Figure 1b), as previously reported [14]. If we used a more cellular damaged *Giardia*, the concentration or signal of gCAD detection could possibly be compromised or degraded by another protease's activity.

### 3.2. gCAD Cloning

With the apoptotic trophozoites of *Giardia lamblia*, we developed an immunoproteomic analysis evidencing the presence of a protein with a MW of 42 kDa and pI 9.4 that was recognized with a commercial polyclonal antibody anti-CAD; this antibody produces cross-reaction between mouse, rat, and human species (Figure 1) [5–7]. In addition, the sequencing derived from the proteomic analysis of this protein, isolated from the spot indicated with the red circle and blue arrow on 2D SDS-PAGE (Figure 1), produced 11 peptides with the following sequences: HSNAELVSPDQIVLR, VAVVVPVSDVEK, IFNSLLADGYFER, LSVTLEEAR, VIAFLTK, VANVPSGVTFDDIK, FKEQNADYLK, AIAESHLQRPR, FINLEER, CAMMAIR, and YQVLDAIREK. In silico analysis demonstrated that the protein identified in the trophozoites of *Giardia lamblia* corresponded to uncharacterized 383 amino acids of A8BHN6 (UniProt) (21 aa more than hCAD), with a theoretical MW of 42 kDa and pI of 9.45 (Figures 1, 1, and 1). These findings support the identification of the gCAD.

### 3.3. Recombinant gCAD

**Figure 2:**
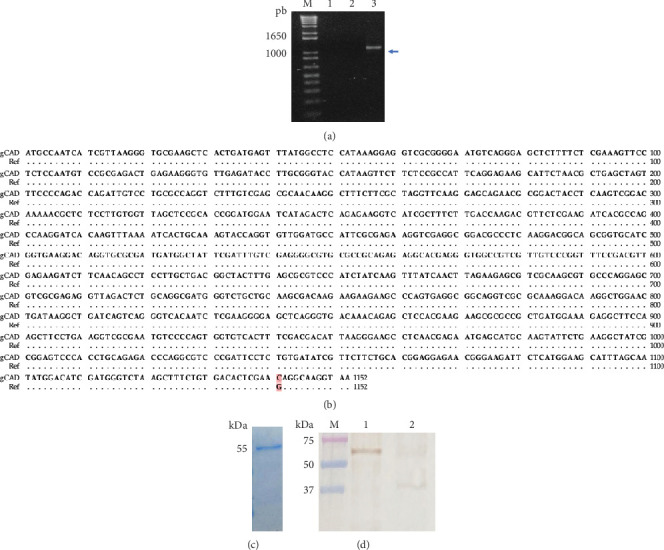
Cloning and recombinant expression of gCAD. (a) Full-length *gCAD* gene amplified by PCR. M, DNA ladder 1 kb; line 1, mock PCR; Line 2, PCR with irrelevant genomic DNA from *Salmonella* Typhimurium as negative control; Line 3, PCR with genomic DNA from *Giardia lamblia*. The *gCAD* gene is indicated by a blue arrow. (b) DNA sequence comparison of the full-length *gCAD* gene (1152 bp) with *hCAD* (reference). The only difference in 1141 is highlighted in red. (c) Recombinant gCAD (rgCAD). rgCAD was purified using nickel-affinity chromatography from solubilized inclusion bodies from *Escherichia coli* expressing rgCAD and visualized on SDS-PAGE 12% as a 55 kDa band. (d) rgCAD is recognized by the polyclonal anti-CAD. Line M: molecular weight; Line 1, rgCAD (55 kDa); Line 2, gCAD (42 kDa). The results are representative of three independent experiments.

To continue studying the gCAD and increase the accuracy of our findings obtained in immunoproteomic analysis, we performed a specific PCR to clone the full-length *gCAD* gene, obtaining a band with 1152 bp (Figure 2a), which was then isolated, sequenced, and deposited in GenBank with the Number ON707040. The alignment match was 99.91% with the *Giardia lamblia* sequence in GenBank XM_001706846.1, with only one difference, C1141G (Figure 2b), localized on Chromosome 3 of *Giardia lamblia* WB. Afterward, the *gCAD* gene was ligated in pET SUMO and was used to produce its recombinant protein in *Escherichia coli*, which was purified and visualized by SDS-PAGE as a fusion protein with a 55-kDa conformation of gCAD (42 kDa) and sumo protein (13 kDa) (Figure 2c). This rgCAD retained its antigenicity, as shown by Western blotting analysis, when compared with native gCAD using the commercial polyclonal anti-CAD (Figure 2d). In addition, these results reinforce the notion that we cloned and expressed gCAD as a recombinant protein, which is a basis for other analyses of apoptosis in *Giardia lamblia*.

### 3.4. 3D Structure of gCAD

**Figure 3:**
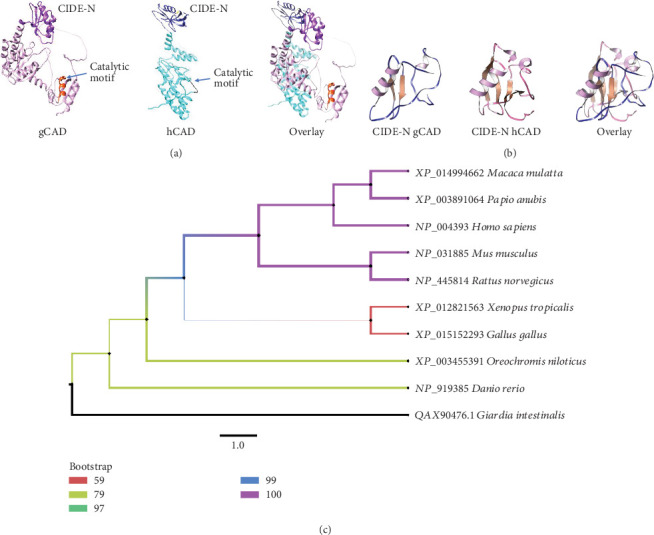
gCAD is an orthologous form of hCAD. (a) Comparison of the 3D structure of gCAD and hCAD. In gCAD, the CIDE-N domain (purple) and putative catalytic motif HASILKAIAESH (red) are indicated. The CIDE-N domain (dark blue) and catalytic motif HKKTTH (black) are indicated. In the overlay, these structures show some conformational similarities. (b) The 3D structure of the CIDE-N domain of gCAD and CIDE-N domain of hCAD (PDB ID: 1IBX). In the overlay, the structural similarities as the *α*-helix and *β*-sheet suggest the potential interaction with an inhibitor in gCAD. (c) Localization of gCAD on phylogenetic tree compared with other reported CAD proteins. NP_001247612 (*Macaca mulatta*), XP_003891142.1 (*Papio anubis*), NP_004392 (*Homo sapiens*), NP_001020467 (*Mus musculus*), XP_417610 (*Gallus gallus*), XP_002939454 (*Xenopus tropicalis*), XP_003444949 (*Oreochromis niloticus*), NP_001002631 (*Danio rerio*), and gCAD QAX90476.1 (*Giardia lamblia*). The tree (neighbor-joining) was derived based on the alignment of the complete amino acid sequences, and the node values represent the percentage of the bootstrap confidence level derived from 1000 replicates. The bar indicates the genetic distance.

The proteomic profile and sequence of *gCAD* and its recombinant protein supported a structural and phylogenetic analysis. The aminoacidic gCAD sequence exhibited 20.93% identity with hCAD alignment; importantly, gCAD contains the CIDE-N-terminal domain (Figure 3), which was previously identified in hCAD [19]. On the other hand, the C-terminal analysis of gCAD showed the presence of a possible catalytic domain H-K-H (residues 326-331-337) (Figure 3), which has been reported in CAD [7]. Finally, the phylogenetic tree obtained from eight additional species revealed that gCAD was the most distant ancestor of all CAD orthologs analyzed, with at least three nodes of separation (Figures 1 and 3). All eukaryotic cells included in the original *Giardia lamblia* cell line suffer from death in an apoptosis-like manner with the participation of DNases like CAD. The identification of gCAD is an important advance in *Giardia lamblia* biology; however, its enzymatic activity should be demonstrated.

## 4. Discussion

In general, in eukaryotic cells with less complexity, the apoptosis signaling pathway can be divided into two pathways: the caspase-dependent or mitochondria-dependent pathway, including some protozoa such as *Trypanosoma brucei* [12, 24]. However, in other protozoa without mitochondria, such as *Giardia lamblia*, the apoptosis signaling pathway uses a caspase-like–dependent pathway [12, 24]. Bagchi et al. could not detect caspase activity or identify any genes involved in apoptosis when analyzing the programmed cell death in *Giardia lamblia* induced by metronidazole or H_2_O_2_ [12]. Notwithstanding, these authors and others observed cellular apoptosis–like behavior through PS expression and genomic DNA degradation (DNA-ladder assay) using metronidazole or curcumin as an alternative pharmacological therapy to kill *Giardia lamblia* in vitro [14]. These reports, along with established knowledge of the execution step that ends with DNA degradation, indicate the presence of a CAD orthologous protein in *Giardia lamblia* [1, 9, 14].

Taking advantage of the cross-reactivity properties of some antibodies, especially polyclonal, some noteworthy examples include the COVID-vaccine protection against different SARS-CoV2 stains [25]; we acquired commercial polyclonal antibodies that recognize mouse, rat, and human CAD and used them to identify gCAD in apoptotic *Giardia lamblia* trophozoites in a similar procedure as described by Pérez-Arriaga et al. [14]. First, *Giardia lamblia* apoptosis analysis was observed in the PS extracellular expression staining with annexin V conjugated to fluorescence (Alexa flour) and defined 3 *μ*M as the better concentration of curcumin that preserves the *Giardia lamblia* piriform morphology (Figure 1a). This data was consistent with Perez-Arriaga et al. [14], who observed that higher concentrations of curcumin result in an altered or a disrupted cellular membrane, findings that suggest necrosis. Using these conditions and the commercial anti-CAD and immunoproteomic protocols described by us previously [17, 26], we identified an antigenic spot with biochemical parameters of MW 42 kDa and pI 9.4, similar to hCAD [15] (Figure 1c,d). When the spot was sequenced, 11 peptides were obtained in the proteomic profile, supporting the discovery of gCAD. Moreover, the gCAD sequence exhibited 22.43% and 23.96% aminoacidic identity with hCAD (Figure 1e) and mCAD, respectively. Although the homology percentage was less than 40%, we were able to identify critical CAD functional regions such as the CIDE-N domain, whose role in the interaction with ICAD prevents spontaneous DNA degradation and inhibits *Giardia lamblia* death, a process that also occurs in other eukaryotic cells [1, 5, 9, 15]. Another potential catalytic motif identified was H-K-H which mediates the nuclease activity in the carboxy-terminal localized in residues 326-331-337 (highlighted in yellow on Figure 1e). This finding was consistent with Woo et al. [7], who proposed that the H-K-H motif is critical for the active catalytic domain.

In fact, bioinformatics focused on 3D protein comparative structure analysis is becoming more accessible using free software, accessible databases such as PDB, and specialized websites online. Using some of the methods described in Section 2.6, we observed the *α*/*β* roll structure with two *α*-helices and two *β*-strands in the CIDE-N domain of gCAD (as seen in the crystal structure of CIDE-N domain of FSP27) [27] and the hCAD NMR structure (PDB ID: 1IBX). As observed in the primary structure of gCAD (Figure 2e) and described above, the possible motif H-K-H was localized in the residues 326-331-337 in the C-terminal region of gCAD. However, structural comparison with hCAD [7] revealed differences in localization and secondary structure, possibly due to a longer aminoacidic gCAD sequence and the phylogenetic distance observed between the sequences analyzed of CAD orthologues. To confirm whether this difference affects the catalytic DNase activity in gCAD, more studies should be conducted. To continue analyzing the key role of gCAD in the apoptosis signaling pathway, we cloned its gene and expressed it as a recombinant 6-His-tag protein in a prokaryotic system and purified it using a previously reported standardized biotechnological strategy [18]. This rgCAD retained its antigenic properties and can be used to produce specific antibodies that will offer another option to try to isolate the orthologous ICAD. Furthermore, this could be used to evaluate DNase activity or conduct structural crystallography analysis, advancing the knowledge of apoptosis or cellular death in *Giardia lamblia*, highlighting similarities and differences with a more evolved eukaryotic cell.

## 5. Conclusions

gCAD was immunoproteomically identified, cloned, and expressed as a recombinant protein. Our data on gCAD presented here represents the first evidence of this orthologous to human CAD. These findings will facilitate further studies of the apoptosis signaling pathway in *Giardia lamblia*.

## Data Availability

Data is available at https://www.ncbi.nlm.nih.gov/gene/?term=ON707040.

## References

[1] Elmore S. (2007). Apoptosis: a review of programmed cell death. *Toxicologic Pathology*.

[2] Kerr J. F. R., Wyllie A. H., Currie A. R. (1972). Apoptosis: a basic biological phenomenon with wideranging implications in tissue kinetics. *British Journal of Cancer*.

[3] Banesh S., Trivedi V. (2021). CD36 ectodomain detects apoptosis in mammalian cells. *Molecular Biotechnology*.

[4] Bossy-Wetzel E., Green D. R. (2000). Detection of apoptosis by annexin V labeling. *Methods in Enzymology*.

[5] Larsen B. D., Sørensen C. S. (2017). The caspase‐activated DNase: apoptosis and beyond. *The FEBS Journal*.

[6] Miles M. A., Hawkins C. J. (2017). Executioner caspases and CAD are essential for mutagenesis induced by TRAIL or vincristine. *Cell Death and Disease*.

[7] Woo E.-J., Kim Y.-G., Kim M.-S. (2004). Structural mechanism for inactivation and activation of CAD/DFF40 in the apoptotic pathway. *Molecular Cell*.

[8] Wang X., Wang X., Cao J. (2023). Environmental factors associated with *Cryptosporidium* and *Giardia*. *Pathogens*.

[9] Navarro M. F., Salvesen G. (2016). Apoptosis. *Encyclopedia of Cell Biology*.

[10] Ahmed M. (2023). Intestinal parasitic infections in 2023. *Gastroenterology Research*.

[11] Minetti C., Chalmers R. M., Beeching N. J., Probert C., Lamden K. (2016). Giardiasis. *BMJ*.

[12] Bagchi S., Oniku A. E., Topping K., Mamhoud Z. N., Paget T. A. (2012). Programmed cell death in *Giardia*. *Parasitology*.

[13] Vanacova S., Liston D. R., Tachezy J., Johnson P. J. (2003). Molecular biology of the amitochondriate parasites, *Giardia intestinalis*, *Entamoeba histolytica* and *Trichomonas vaginalis*. *International Journal for Parasitology*.

[14] Pérez-Arriaga L., Mendoza-Magaña M. L., Cortés-Zárate R. (2006). Cytotoxic effect of curcumin on *Giardia lamblia* trophozoites. *Acta Tropica*.

[15] Liu X., Li P., Widlak P. (1998). The 40-kDa subunit of DNA fragmentation factor induces DNA fragmentation and chromatin condensation during apoptosis. *Proceedings of the National Academy of Sciences*.

[16] Eckhart L., Fischer H., Tschachler E. (2007). Phylogenomics of caspase-activated DNA fragmentation factor. *Biochemical and Biophysical Research Communications*.

[17] Piedra-Quintero Z. L., Apodaca-Medina A. I., Beltrán-López E. (2015). Immunoproteomic identification of P29 antigen as the elongation factor-1*α* of *Leishmania mexicana*. *Vector-Borne and Zoonotic Diseases*.

[18] López-López K., Apodaca-Medina A. I., León-Sicairos C. R. (2018). Cloning and recombinant expression of elongation factor-1*α* of Leishmania mexicana. *Vector-Borne and Zoonotic Diseases*.

[19] Katoh K., Kuma K., Toh H., Miyata T. (2005). MAFFT version 5: improvement in accuracy of multiple sequence alignment. *Nucleic Acids Research*.

[20] Han M. V., Zmasek C. M. (2009). PhyloXML: XML for evolutionary biology and comparative genomics. *BMC Bioinformatics*.

[21] Jumper J., Evans R., Pritzel A. (2021). Highly accurate protein structure prediction with AlphaFold. *Nature*.

[22] Pettersen E. F., Goddard T. D., Huang C. C. (2004). UCSF Chimera - a visualization system for exploratory research and analysis. *Journal of Computational Chemistry*.

[23] Fadok V. A., De Cathelineau A., Daleke D. L., Henson P. M., Bratton D. L. (2001). Loss of phospholipid asymmetry and surface exposure of phosphatidylserine is required for phagocytosis of apoptotic cells by macrophages and fibroblasts. *Journal of Biological Chemistry*.

[24] Chose O., Sarde C. O., Noël C. (2003). Cell death in protists without mitochondria. *Annals of the New York Academy of Sciences*.

[25] Pegu A., O’Connell S. E., Schmidt S. D. (2021). Durability of mRNA-1273 vaccine–induced antibodies against SARS-CoV-2 variants. *Science*.

[26] Salazar-Mejía P. G., Tejeda-Aguirre C. R., López-Moreno H. S. (2010). Reaction of *Leishmania (Leishmania) mexicana* antigens by sera of patients with cutaneous Leishmaniasis from Sinaloa, Mexico. *Salud Pública de México*.

[27] Zhou P., Lugovskoy A. A., McCarty J. S., Li P., Wagner G. (2001). Solution structure of DFF40 and DFF45 N-terminal domain complex and mutual chaperone activity of DFF40 and DFF45. *Proceedings of the National Academy of Sciences of the United States of America*.

[28] López-Moreno H. S., Villa-Medina M. C., Díaz-Gaxiola C. (2023). Cloning and recombinant expression of caspase-activated DNase orthologous gene of Giardia lamblia. *Authorea*.

